# Professional future expectations and mental health in university students: a psychosocial and environmental approach

**DOI:** 10.3389/fpsyg.2026.1815910

**Published:** 2026-10-13

**Authors:** Tania Gaspar, Ana Cerqueira, Fábio Botelho Guedes, Elisa Aires, Catarina N. Matias, Cátia Oliveira, André Tavares Rodrigues, Inês Jongenelen

**Affiliations:** 1 HEI-Lab – Digital Human-Environment Interaction Labs/SPIC – Psychology, Innovation and Knowledge Service, Lusófona University, Lisbon, Portugal; 2 Environmental Health Institute (ISAMB), Faculty of Medicine, University of Lisbon (FMUL), Lisbon, Portugal; 3 Portuguese Lab for Healthy Workplaces (LABPATS)/Aventura Social Association (ASA), Lisbon, Portugal; 4 CIDEFES – Lusófona University and CIFI2D – University of Porto, Porto, Portugal; 5 Center for Psychology, Faculty of Psychology and Educational Sciences, University of Porto, Porto, Portugal; 6 Lusófona Education – ISMAT – Manuel Teixeira Gomes Institute, Portimão, Portugal

**Keywords:** ecological model, health promotion, higher education, mental health, professional future expectations

## Abstract

**Introduction:**

University students face heightened risks regarding mental health, with depression, anxiety, and stress being the most prevalent issues. Students’ mental health is associated with their academic performance and psychosocial well-being, as well as with their development and future professional trajectories.

**Methods:**

This study is part of the Iberian Observatory of Healthy Learning and Youth Participation (OIASP-J) and uses the Healthy Learning Environments Ecosystems instrument. The sample consisted of included 2,315 university students, of whom 65.5% (*n* = 1,516) were female, with a mean age of 22.9 years (*SD* = 5.18).

**Results:**

Older male students who value institutional ethics and values, demonstrate greater academic engagement, report higher levels of well-being, more effective stress management, and greater happiness, exhibit fewer psychosocial risks related to well-being and mental health. Social isolation emerged as a significant factor associated with greater vulnerability in well-being and mental health. Regarding professional future expectation, students with more positive expectations exhibited better individual, psychosocial, and organizational indicators than those with more negative expectations.

**Conclusion:**

We conclude that the results provide evidence for higher education institutions, health professionals, and policymakers to invest in more skills programs and mental health interventions. These programs may help students thrive academically, maintain good mental health throughout their studies, and successfully complete their training. In turn, this facilitates a smooth transition into professional life and enables students to contribute to society.

## Introduction

The ecological model of human development has been widely used to understand expectations regarding future careers and mental health among university students, as it considers development to result from the dynamic interaction between person, processes, context, and time (Bronfenbrenner, 1979; Bronfenbrenner and Morris, 2006).

The academic trajectory and higher education constitute a central microsystem, in which the most significant proximal interactions occur (e.g., with peers and faculty), closely related to psychological well-being and the construction of life projects. At the same time, this context is interconnected with other systems, such as the mesosystem (e.g., the relationship between university and family), the exosystem (e.g., educational policies or labor market conditions), and the macrosystem (e.g., sociocultural values regarding success and employability), which, independently, shape students’ perceptions of the future and their levels of psychological adjustment (Bronfenbrenner, 1994; Bronfenbrenner and Morris, 2006). Thus, the analysis of professional expectations and mental health within the university context requires an ecological approach that considers not only individual characteristics but also the influence of the multiple contexts in which students are embedded, reinforcing the centrality of the university as a privileged space for development (Bronfenbrenner, 1977; Gaspar et al., 2025; Limone and Toto, 2022; Rosa and Tudge, 2013).

Universities today face new challenges that require the reinvention of their educational models. Research examining students’ perceptions of their preferred learning environments has identified several key themes shaping contemporary higher education, including institutional characteristics, availability of resources, flexibility and learning opportunities, pedagogical approaches, and the use of information and communication technologies (Valtonen et al., 2021). Importantly, evidence suggests that the organizational environment is associated various aspects of students’ functioning, including mental health, resilience, and academic engagement (Norabuena-Figueroa et al., 2025; Wang et al., 2025).

Mental health issues among university students are a prominent topic in the literature, with an increasing number of students experiencing psychological distress (Dessauvagie et al., 2022; Gardani et al., 2022; Gaspar et al., 2024a; Limone and Toto, 2022; Porru et al., 2021). Depression, anxiety, and stress are the most common problems (Duraku et al., 2023; Hossain et al., 2022; Quincho et al., 2021; Tan et al., 2023). A range of academic, social, psychological, and lifestyle-related factors has been identified as associated to these difficulties, highlighting the complex interplay of factors related to students’ mental health and well-being (Limone and Toto, 2022). It is estimated that approximately 75% of all psychological disorders begin before the age of 24, further emphasizing the vulnerability of university students (Amaro et al., 2024). This increased vulnerability highlights the limitations of mental health services, which are evident in the healthcare system and academic institutions (Limone and Toto, 2022).

As is well known, higher education students face numerous stressors that often exceed their coping resources (Maymon and Hall, 2021; Olivera et al., 2023; Renn and Reason, 2013). Factors such as leaving the family home, academic difficulties, pressure to succeed, uncertainties about life after university, and dysfunctional social relationships (e.g., competitiveness, bullying) are frequently associated with students’ mental health (Amaro et al., 2024; Bersia et al., 2024).

The literature indicates that the transition from secondary to higher education is often accompanied by increased stress and anxiety, placing first-year students at greater risk of mental illness. In this regard, strong social support is widely recognized as an important factor for higher education students, regardless of their year of study (Campbell et al., 2022; McLean et al., 2023; Maymon and Hall, 2021; Olivera et al., 2023).

Students’ mental health is significantly associated with their academic performance, shaping their development and future professional trajectories (Wenjuan et al., 2020). During higher education, students encounter important decisions regarding their careers. This period is also marked by feelings of uncertainty, which contribute to increased anxiety and stress. A lack of adequate coping mechanisms, weak support networks, or experiences of isolation can exacerbate psychological symptoms, underscoring the complex interplay between the academic, personal, and social challenges that students at this stage face (Guzmán-Utreras et al., 2023; Wang J. et al., 2023).

Gender differences in university students’ mental health have been widely studied (Wenjuan et al., 2020). Research consistently shows that female students are more likely to experience psychological distress, particularly anxiety and depression, making them a higher-risk group in this area (Amaro et al., 2024; Bersia et al., 2024; Hossain et al., 2022; Porru et al., 2021; Rubach et al., 2022; Teixeira et al., 2022; Wenjuan et al., 2020). For example, Nogueira et al. (2022) found that among first-year Portuguese university students, female students showed poorer mental health indicators, greater dissatisfaction with academic performance, higher psychological vulnerability, and increased exposure to psychosocial risks. Similarly, studies based on the Health Behavior in School-aged Children (HBSC) survey indicate that during adolescence, girls report lower well-being and more psychological symptoms than boys (Gaspar et al., 2022; Gaspar et al., 2024a).

Regarding age differences, some studies have noted that well-being and mental health tend to decline with increasing age, particularly during periods of major transition. Higher education constitutes one such transition, during which students face greater academic demands, increased responsibilities, and uncertainties about their personal and professional futures (Barbayannis et al., 2022; Douwes et al., 2023; Sivertsen et al., 2023).

In the face of uncertainty across various areas of their lives, young people develop expectations about their future. They may anticipate that their future will improve, remain stable, or deteriorate (An and Suh, 2025). Findings from Ma and Shea (2021) indicate that university students’ future expectations are associated with perceived educational and career barriers. Moreover, contextual factors, particularly the sense of belonging within the university environment, play a crucial mediating role in this relationship. Furthermore, the authors identified university students’ expectations of professional success as a factor associated with motivation and academic performance, noting that these expectations are also influenced by sociodemographic characteristics such as gender (Fernández et al., 2023).

The transition to adulthood involves a wide range of challenges and expectations that, when misaligned with students’ actual lived experiences – expectations shaped not only by students themselves but also by their families – can be associated with psychological distress and increase risks to university students’ mental health and well-being (Culatta and Clay-Warner, 2021). In this context, personal experiences and social environments are key factors linked to young people’s expectations about their futures, thereby influencing their emotional adjustment and academic engagement in higher education (An and Suh, 2025). In this study, the concept of professional future expectations is used to operationalize dimensions related to confidence, entrepreneurial ambition, labour mobility, and income expectations (Gaspar et al., 2023a; Gaspar et al., 2023b; Gaspar et al., 2024b; Gaspar et al., 2025). The examination of institutional and relational aspects associated with educational environments is framed within large-scale ecological studies that investigate psychosocial environments, health, and well-being (Gaspar et al., 2023a; Gaspar et al., 2023b; World Health Organization, 2010).

Beyond the academic context, the professional domain is a central and significant aspect of students’ lives, with significant implications for their well-being and quality of life (Doyle et al., 2023; Wang R. et al., 2023). As they transition into adulthood, young people face important decisions about education, career, and lifestyle. Nowadays, they have greater freedom and a broader range of options to define their personal and professional trajectories and choices (Doyle et al., 2023). Consequently, future expectations constitute an important dimension of students’ personal and social development with significant associations reported for well-being, motivation, decision-making, performance, and mental health (An and Suh, 2025; Erçetin et al., 2020; Gönen and Öktem, 2022). Considering the above and grounded in an ecological perspective, which highlights the interplay between individual characteristics and contextual factors, the present study aims to examine the relationship between university students’ professional future expectations (high versus low) and sociodemographic, psychological, and organizational variables. It also seeks to understand how these variables are associated with psychosocial risks affecting well-being and mental health in the university context.

## Methods

### Participants

This study included 2,315 university students, of whom 65.5% (*n* = 1.516) were female, with a mean age of 22.93 years (*SD* = 5.18). The sample consisted of full-time students (62.7%), working students (37.2%), and Erasmus students (0.1%). Most students (76.3%) were residents already living in the institution’s region, while 20.7% were living away from home within the country, and 3.1% were living abroad.

### Variables and measures

In line with the study objectives, the variables presented in Table 1 were considered.

**Table 1:** Variables and measures under study.

Variables	Measures
Age	Continuous variable, expressed in years, corresponding to each participant’s age.
Gender	1 – Female; 2 – Male
Professional future expectations^1^	Scale consisting of 6 items, using a 5-point Likert scale. Scores may range from 1 to 5, with higher values indicating poorer expectations about the professional future (e.g., When I complete my higher education, I will be prepared to manage and build my professional future; I believe I will be able to have good employment opportunities). *α* = 0.81 Recoded as: 1 – High professional future expectations; 2 – Low professional future expectations. Recoding was performed based on percentiles, with responses at or below the 25th percentile classified as “low professional future expectations.” This dichotomization approach is consistent with previous studies that have classified individuals in the lowest quartile as a distinct low-outcome group (e.g., Jerrim, 2014; Plenty and Mood, 2016).
Ethics and values^2^	Scale with 3 items, rated on a 5-point Likert scale from 1 “strongly disagree” to 7 “strongly agree.” Higher scores indicate lower risk associated with the values and relational practices adopted by the higher education institution, reflected in students’ safety and health. *α* = 0.87
Students engagement^2^	Scale with 3 items, rated on a 5-point Likert scale from 1 “strongly disagree” to 7 “strongly agree.” Higher scores indicate lower risk associated with students’ sense of belonging, participation, and integration within the institution. *α* = 0.84
Psychosocial environment^2^	Scale with 6 items, rated on a 5-point Likert scale from 1 “strongly disagree” to 7 “strongly agree.” Higher scores indicate lower risk associated with the institution’s psychosocial environment, particularly regarding work content and relationships with leadership. *α* = 0.88
Psychosocial risks^2^	Scale with 5 items, rated on a 5-point Likert scale from 1 “strongly disagree” to 7 “strongly agree.” Higher scores indicate a higher risk associated with psychosocial factors of the institution related to well-being and mental health. *α* = 0.87
Well-being^3^	Scale with 5 items, rated on a 6-point Likert scale from 0 “at no time” to 5 “all of the time.” Higher scores indicate lower levels of well-being. *α* = 0.88
Stress management^4^	Scale with 4 items, rated on a 5-point Likert scale from 1 “never/almost never” to 5 “always/almost always.” Higher scores indicate a greater stress management capacity. α = 0.74
Social isolation^5^	Scale with 4 items, rated on a 5-point Likert scale from 1 “never” to 5 “always.” Higher scores indicate higher perceived level of loneliness, reflecting frequent feelings of exclusion, isolation, and lack of companionship. *α* = 0.89
Happiness level^6^	Scale with 4 items, rated on a 7-point Likert scale from 1 “less happy” to 7 “more happy.” Higher scores indicate higher levels of subjective happiness perceived by the individual. *α* = 0.74

^1^LABPATS Protocol, Gaspar et al. (2024b). ^2^Healthy Learning Environment Ecosystems Instrument (EA2S), Gaspar et al. (2023a) and Gaspar et al. (2023b). ^3^HBSC 2022 Protocol, Gaspar et al. (2022). ^4^Cohen et al. (1983). ^5^Russell et al. (1978). ^6^Lyubomirsky and Lepper (1999).

### Procedure

This study is part of a major project by the Iberian Observatory of Healthy Learning and Youth Participation (OIASP-J) of the Portuguese Laboratory for Healthy Work Environments (LABPATS), focusing on healthy learning ecosystems in higher education institutions in Portugal. Learning environments are assessed using a reduced version of the Healthy Learning Environments Ecosystems (EA2S) instrument (Gaspar et al., 2023a; Gaspar et al., 2023b), which is based on the Healthy Workplaces model proposed by the World Health Organization (2010). The survey aimed to assess students’ health, well-being, and learning environmental conditions, with the goal of promoting healthier, more sustainable, and inclusive institutions (Gaspar et al., 2025). Data were collected between 2023 and 2024 from a sample of students from several higher education institutions across Portugal.

Questionnaire responses were collected online and anonymously. The study used a convenience sampling approach, with participants recruited through universities and student associations that disseminated the survey link to students. Confidentiality and anonymity of the data were ensured in accordance with data protection standards. Further details on the study’s data collection procedures can be found in the final report (Gaspar et al., 2025).

The study was approved by the Ethics and Deontology Committee for Scientific Research at the School of Psychology and Life Sciences of Lusófona University of Humanities and Technologies. All participants took part voluntarily, and written informed consent was obtained.

### Data analysis

The data was analyzed using the Statistical Package for the Social Sciences (SPSS), version 28 for iOS. The normality of the data distribution was assessed, and the statistical assumptions were met. The significance level for all statistical procedures was set at alpha 0.05, corresponding to a 95% confidence level. To explore the relationships between the variables in this study, the existing correlations were analysed using Pearson’s correlation coefficient. Descriptive statistics were conducted to characterize the sample. The Chi-square test for independent variables was used to examine the relationship between professional future expectations and gender. Independent samples t-tests were applied to analyze the relationship between professional future expectations and age, ethics and values, student engagement, psychosocial environment, psychosocial risks, well-being, stress management, social isolation, and happiness.

Psychosocial risks related to well-being and mental health, along with the other study variables, were analyzed through three linear regression models, considering sociodemographic factors, organizational factors, and personal factors. Before interpreting the regression models, the main assumptions of linear regression were examined. These included the inspection of normality, linearity, homoscedasticity, independence of errors, and possible multicollinearity among predictors. No relevant violations were identified that would compromise the validity or interpretation of the estimated models. In particular, multicollinearity was assessed using tolerance and variance inflation factor (VIF) values, with acceptable thresholds defined as tolerance values >0.20 and VIF values <5 (Marôco, 2011). Given the applied focus of the study, the results are presented with emphasis on the explained variance, statistical significance, and direction of the associations, while also considering the magnitude and theoretical relevance of the observed effects.

## Results

The analysis of the correlations between the variables in this study (Table 2) shows the existence of statistically significant associations among most of the variables considered. The variables related to the psychosocial context and individual functioning are interrelated, with higher levels of ethics and values, student engagement, and psychosocial environments tending to be associated with better indicators of well-being, stress management, and happiness, as well as lower levels of social isolation.

**Table 2:** Descriptive statistics and correlations between study variables.

Variables	1	2	3	4	5	6	7	8	9
1. Ethics and values	–								
2. Student engagement	0.45***	–							
3. Psychosocial environment	0.66***	0.54***	–						
4. Psychosocial risks	0.49***	0.33***	0.53***	–					
5. Well-being	0.17***	0.26***	0.17***	0.10***	–				
6. Stress management	0.15***	0.31***	0.19***	0.13***	0.30***	–			
7. Social isolation	−0.14***	−0.26***	−0.21***	−0.15***	−0.03	−0.43***	–		
8. Happiness level	0.19***	0.40***	0.25***	0.18***	0.04	0.57***	−0.47***	–	
9. Professional future expectations	0.00	−0.16***	−0.05*	−0.04	−0.08***	−0.11***	0.13***	−0.29***	–

***p < 0.001; *p < 0.05.

On the other hand, social isolation consistently shows negative associations with several variables under analysis, suggesting that higher levels of social isolation are associated with poorer indicators of psychosocial adjustment. Future professional expectations also generally exhibit negative correlations with variables such as engagement, well-being, and happiness, and a positive association with social isolation, indicating that lower expectations tend to coexist with greater social isolation. It is noteworthy that no significant associations were found between ethics and values and future professional expectations, nor between well-being and social isolation and happiness.

The strongest correlations are observed between the psychosocial environment and ethics and values (*r* = 0.66), as well as between stress management and happiness levels (*r* = 0.57). Additionally, the negative association between social isolation and happiness (*r* = −0.47) stands out, reinforcing the relevance of these dimensions for individuals’ adjustment.

Table 3 presents the sample characteristics according to professional future expectations (high versus low). Most participants (71.1%) reported high professional future expectations. Statistically significant differences were observed across all study dimensions. Older students and male students tend to report higher professional future expectations. Students with high professional future expectations scored higher in ethics and values, showed greater engagement, experienced a more positive psychosocial environment related to content and leadership relationships, reported higher well-being, demonstrated better stress management, and indicated higher levels of happiness. In contrast, students with low professional future expectations exhibited greater psychosocial risks related to well-being and mental health, as well as higher social isolation.

**Table 3:** Sample characteristics by levels of professional future expectations (high versus low).

Variables	*M* ± *SD* or % (*n*)			*p*
	Total (*n* = 2,315)	High professional future expectations 71.1% (*n* = 1,287)	Low professional future expectations 28.9% (*n* = 524)	
Age (years)^1^	22.9 ± 5.2	23.1 ± 5.4	22.6 ± 4.7	0.031
Gender^2^				0.017
Male	34.5 (799)	37.4 (480)*	31.4 (161)	
Female	65.5 (1,516)	82.6 (803)	68.6 (351)*	
Ethics and values^1^	3.42 ± 0.86	3.46 ± 0.87	3.19 ± 0.90	≤0.001
Student engagement^1^	3.68 ± 0.89	3.85 ± 0.86	3.33 ± 0.99	≤0.001
Psychosocial environment^1^	3.65 ± 0.78	3.74 ± 0.78	3.43 ± 0.81	≤0.001
Psychosocial risks^1^	3.31 ± 1.12	3.23 ± 1.12	3.54 ± 1.11	≤0.001
Well-being^1^	6.78 ± 1.63	7.07 ± 1.47	6.16 ± 1.75	≤0.001
Stress management^1^	3.13 ± 0.76	3.24 ± 0.75	2.84 ± 0.78	≤0.001
Social isolation^1^	2.39 ± 0.76	2.32 ± 0.92	2.59 ± 0.95	≤0.001
Happiness level^1^	4.69 ± 1.18	4.88 ± 1.10	4.17 ± 1.25	≤0.001

^1^Independent sample *t*-test; ^2^Chi-square. *Adjusted residuals >1.96. M, Mean; SD, standard deviation.

Table 4 presents three multiple linear regression models analyzing the sociodemographic, organizational, and personal factors that potentially explain psychosocial risks in higher education institutions affecting well-being and mental health.

**Table 4:** Multiple linear regression model of variables predicting the psychosocial risks in the educational institution related to well-being and mental health.

Models	Variables	High professional future expectations				Collinearity statistics		Low professional future expectations				Collinearity statistics	
		*B*	Standard error	Βeta	*t*	Tole-rence	VIF	*B*	Standard error	Βeta	*t*	Tole-rence	VIF
Model 1 – Sociodemographic factors	(Constant)	4.18	0.12		34.27***			4.39	0.19		23.07***		
	Age	−0.02	0.00	−0.14	−5.24***	1.00	1.00	−0.02	0.01	−0.10	−2.33*	1.00	1.00
	Gender	−0.31	0.05	−0.17	−6.36***	1.00	1.00	−0.36	0.07	−0.21	−4.92***	1.00	1.00
		*R*^2^ = 0.049; *F* = 34.194 (2/1,286), *p* < 0.001						*R*^2^ = 0.050; *F* = 14.755 (2/523), *p* < 0.001					
Model 2 – Organizational factors	(Constant)	5.14	0.16		32.06***			4.99	0.23		22.08***		
	Age	−0.02	0.00	−0.10	−3.87***	0.94	1.07	−0.01	0.01	−0.06	−1.31	0.97	1.03
	Gender	−0.31	0.05	−0.17	−6.50***	0.98	1.02	−0.35	0.07	−0.21	−4.95***	0.98	1.02
	Ethics and values	−0.06	0.04	−0.06	−1.50	0.56	1.79	−0.10	0.06	−0.11	−1.74	0.42	2.39
	Student engagement	−0.17	0.03	−0.17	−5.38***	0.67	1.50	−0.17	0.04	−0.22	−4.30***	0.58	1.71
	Psychosocial environment	−0.07	0.04	−0.06	−1.59	0.49	2.05	0.03	0.06	0.03	0.45	0.35	2.83
		*R*^2^ = 0.107; *F* = 31.935 (5/1,286), *p* < 0.001						*R*^2^ = 0.115; *F* = 14.611 (5/523), *p* < 0.001					
Model 3 – Personal factors	(Constant)	4.79	0.25		19.56***			5.04	0.32		15.66***		
	Age	−0.01	0.00	−0.07	−2.81**	0.91	1.10	−0.01	0.01	−0.04	−1.12	0.92	1.09
	Gender	−0.17	0.04	−0.10	−4.03***	0.92	1.09	−0.26	0.06	−0.15	−4.05***	0.92	1.08
	Ethics and values	−0.08	0.03	−0.08	−2.36*	0.55	1.82	−1.12	0.05	−0.14	−2.53**	0.41	2.46
	Student engagement	−0.07	0.03	−0.07	−2.40*	0.61	1.63	−0.06	0.04	−0.07	−1.46	0.47	2.15
	Psychosocial environment	−0.03	0.04	−0.03	−0.82	0.49	2.06	0.04	0.05	0.05	0.81	0.34	2.93
	Well-being	0.13	0.02	0.14	5.63***	0.98	1.02	0.11	0.03	0.17	3.87***	0.94	1.06
	Stress management	−0.23	0.03	−0.27	−10.00***	0.63	1.60	−0.25	0.05	−0.25	−5.33***	0.48	2.07
	Social isolation	0.23	0.03	0.19	7.33***	0.74	1.35	0.14	0.05	0.12	2.80**	0.63	1.59
	Happiness level	−0.18	0.03	−0.18	−6.46***	0.61	1.65	−0.21	0.04	−0.24	−5.27***	0.49	2.04
		*R*^2^ = 0.328; *F* = 70.606 (9/1,286), *p* < 0.001						*R*^2^ = 0.319; *F* = 28.235 (9/523), *p* < 0.001					

*p ≤ 0.05; **p ≤ 0.01; ***p ≤ 0.001.

Model 1, which includes sociodemographic factors, explains 4.9% of the variance for high professional future expectations, *F*(2, 1,286) = 34.194; *p* ≤ 0.001 and 5.0% of the variance for low professional future expectations, *F*(2, 523) = 14.755; *p* ≤ 0.001. In both groups, age was negatively associated with psychosocial risks, indicating that older students exhibit fewer psychosocial risks than younger students (as age increases, psychosocial risks slightly decrease). Gender was also significant, showing a negative association, with male students presenting lower psychosocial risks related to mental health compared to female students.

Model 2, which focuses on organizational factors, explains 10.7% of the variance in psychosocial risks related to mental health for the group with high professional future expectations, *F*(5, 1,286) = 31.935; *p* ≤ 0.001 and 11.5% for the group with low professional future expectations, *F*(5, 523) = 14.611; *p* ≤ 0.001. Among students with high professional future expectations, those who are older, male, and more engaged in the institution exhibit lower psychosocial risks related to well-being and mental health. In the group with low professional future expectations, male students and those with higher engagement similarly show lower psychosocial risks related to well-being and mental health. These models indicate that, beyond sociodemographic characteristics, student engagement is an important organizational factor for both groups (high or low professional future expectations) regarding the psychosocial risks related to well-being and mental health.

Model 3, which adds personal factors to the sociodemographic and organizational variables, explains 32.8% of the variance in psychosocial risks for students with high professional future expectations, *F*(9, 1,286) = 70.606; *p* ≤ 0.001 and 31.9% for students with low professional future expectations, *F*(9, 523) = 28.235; *p* ≤ 0.001. In the group of students with high professional future expectations, being older and male, valuing the institution’s ethics and values, demonstrating greater academic engagement, higher levels of well-being, better stress management, and higher levels of happiness were all associated with lower psychosocial risks related to well-being and mental health. Social isolation emerged as a significant factor, being associated with greater vulnerability in well-being and mental health.

Among students with low professional future expectations, being male, valuing institutional ethics and values, having higher well-being, better stress management, and greater happiness are associated with a lower likelihood of psychosocial risks related to well-being and mental health. These results highlight that personal factors such as promoting well-being, managing stress, fostering happiness, and reducing social isolation are the main factors associated with psychosocial risks related to students’ mental health, regardless of whether their professional future expectations are high or low.

## Discussion

### Global interpretation of the results

The aim of this study was to analyze the relationship between university students’ professional future expectations (high versus low), sociodemographic, psychological, and organizational variables. The study also sought to understand how these variables are associated with psychosocial risks related to well-being and mental health in the university context.

The results highlight a strong relationship between university students’ professional future expectations and their psychosocial well-being. Students with high future expectations exhibit significantly better indicators of mental health and academic engagement as they place greater value on institutional ethics and values, demonstrate higher involvement in academic activities, and report greater subjective well-being, better stress management, and higher levels of happiness. In contrast, students with low future expectations show greater psychosocial vulnerability, including lower well-being, higher prevalence of psychological distress symptoms, and a tendency toward greater social isolation.

When analyzing psychosocial risks related to mental health among university students, and comparing different levels of professional future expectations, the results indicate that age and gender have a statistically significant, although relatively modest, association. When sociodemographic, psychosocial, and organizational variables are included in the model, they explain approximately one-third of the variance in psychosocial risks related to mental health. Psychosocial factors such as well-being, stress management, happiness, and social isolation emerge as the main factors associated with these risks, regardless of students’ levels of professional future expectations.

### Role of individual and institutional factors

Indicators of mental health among higher education students have declined in recent years, with increases in symptoms of anxiety and depression and decreases in positive factors such as perceived happiness and life satisfaction. The observed association between individual characteristics and psychosocial risks, independent of students’ expectations about their professional future, is in line with previous literature (Duraku et al., 2023; Hossain et al., 2022; Limone and Toto, 2022; Maymon and Hall, 2021; Quincho et al., 2021; Tan et al., 2023).

An important aspect of the findings is that organizational variables differentiate students with high and low future expectations, even though personal psychosocial variables show stronger associations for mental health vulnerability. This suggests that institutional contexts may indirectly be related to engagement, belonging, emotional regulation, and future orientation. Recent literature increasingly conceptualizes universities as social ecosystems in which institutional culture, perceived fairness, participation, relational climate, and organizational legitimacy are associated with students’ capacity to cope with uncertainty and transition-related stressors (Cayubit, 2022; Ekornes, 2022; Fang et al., 2025; Kassab et al., 2024; Pawlak and Moustafa, 2023; Tecilazić et al., 2026). Therefore, institutional culture may shape the conditions under which students develop adaptive psychosocial resources, while proximal individual factors remain more directly associated with mental health vulnerability. These findings align with socioecological perspectives that emphasize the interaction between individual and institutional conditions (Bronfenbrenner, 1977; Gaspar et al., 2025; Limone and Toto, 2022; Rosa and Tudge, 2013; Thaivalappil et al., 2023).

### Professional future expectations as an indicator

Students reporting more positive future expectations presented higher levels of well-being, emotional competencies, academic satisfaction, and perceived social support, whereas lower expectations were associated with indicators of mental health risk, including psychological distress and social isolation (Ma and Shea, 2021). Therefore, promoting a positive outlook on the future, along with strengthening associated personal and social resources, may contribute to enhancing students’ well-being and reducing psychosocial risks associated with low professional future expectations (Bersia et al., 2024; Dessauvagie et al., 2022; Duraku et al., 2023; Gardani et al., 2022; Gaspar et al., 2024a; Limone and Toto, 2022; Porru et al., 2021).

Future expectations may be considered a psychosocial marker associated with students’ capacity to navigate academic, social, and professional transitions. Lower expectations may not simply indicate pessimism regarding career prospects, but may also be associated with emotional overload, social disconnection, and reduced perceived agency within the university context (Latorre-Cosculluela et al., 2022; Mamani-Benito et al., 2023; Picker-Roesch and Lang, 2024).

The results also indicate differences according to gender and age, reflecting variation in psychosocial experiences across student groups and reinforcing the importance of considering heterogeneity when designing interventions to support students’ needs. Evidence suggests that female students tend to internalize psychological distress, whereas male students are more likely to externalize it. Additionally, gendered social expectations, such as the tendency for girls to be encouraged toward diligence and responsibility, may contribute to higher levels of anxiety and greater difficulty in managing both self-imposed and external expectations (Amaro et al., 2024; Bersia et al., 2024; Gaspar et al., 2024a; Hossain et al., 2022; Wenjuan et al., 2020).

### Practical implications for promoting a healthy psychosocial environment and mental health in higher education institutions

This study contributes to research in psychology and the educational sciences in higher education, as well as to institutional practice and mental health policies targeting university students. From a scientific perspective, the findings enhance understanding of how students’ professional future expectations relate to psychological well-being and academic functioning. They also support evidence from positive psychology and academic motivation literature indicating that optimism and related psychological resources are associated with more favorable adjustment outcomes, including higher well-being and lower emotional difficulties (Forgeard, 2012).

From an applied perspective, the results highlight relevant areas for intervention in higher education settings. The identification of factors, such as well-being, stress regulation, happiness, and alignment with institutional values, suggests that universities and support services should implement initiatives to strengthen these dimensions. These may include well-being programs, socioemotional skills training, accessible psychological support, and activities aimed at fostering belonging and reducing social isolation. Given the association between social isolation and mental health risk, peer support networks and mentoring systems may be particularly relevant in addressing psychological distress (Campbell et al., 2022; McLean et al., 2023; Maymon and Hall, 2021; Olivera et al., 2023).

These interventions are also likely to have broader academic and institutional benefits. Improved mental health is associated with better academic performance and persistence, whereas psychological distress has been linked to lower achievement and higher dropout risk (Guzmán-Utreras et al., 2023; Wang J. et al., 2023). In this sense, supporting students’ mental health may be associated with retention and completion rates, with potential downstream implications for educational and workforce outcomes. Recent data suggest that providing adequate well-being support can enhance institutional success rates, which may contribute to preparing future professionals who are healthier, more resilient, and more engaged as citizens. Ultimately, these benefits extend beyond higher education, with potential implications for the economy and society, including contributions to socio-economic growth (Norabuena-Figueroa et al., 2025; Wang et al., 2025).

At the institutional level, evidence indicates that institutional attitudes, administrative (in)action, perceived campus safety, and the overall organizational climate are linked to students’ mental health, highlighting the importance of university-wide strategies that complement individual-level interventions (Thaivalappil et al., 2023). Accordingly, integrated approaches combining student-focused support with institutional policies that promote a healthy, inclusive, and supportive academic environment are recommended (Norabuena-Figueroa et al., 2025; Wang et al., 2025).

### Limitations

The present study has some limitations that should be considered when interpreting the results. First, the use of a convenience sample may limit the generalizability of the findings. Although this study provides relevant evidence on the relationship between professional future expectations, psychosocial variables, and mental health-related risks, the exclusive use of self-report measures collected at a single time point should be acknowledged as a methodological limitation. This may have increased the possibility of common method bias and may partly explain the magnitude of some of the observed associations, particularly between conceptually related psychosocial variables. Therefore, the results should be interpreted as associations rather than causal effects. Future research should consider longitudinal designs, multiple informants, mixed-method approaches, or objective institutional indicators to reduce shared method variance and strengthen the robustness of the findings.

The dichotomization of professional future expectations should also be considered when interpreting the results. Although this analytical option was useful for identifying psychosocial profiles associated with higher and lower expectations, it inevitably involved a reduction of variability in the original measure. In addition, the distribution of participants across groups was unequal, with 71.1% of students classified as having high professional future expectations. This imbalance may have influenced the comparison between groups and limits the interpretation of the findings as reflecting two equivalent subgroups. Therefore, the results should be understood as indicating broad psychosocial patterns associated with more positive versus less positive professional future expectations, rather than as evidence of clearly distinct categories. Future studies may benefit from examining professional future expectations as a continuous variable or by using alternative person-centered approaches. Despite these limitations, the study also presents several strengths: it is a large-scale study, follows a rigorous methodology consistently applied by all LABPATS partners, and allows for comparisons across all studies using this protocol.

## Conclusion

In conclusion, this study highlights the importance of addressing university students’ mental health through an ecological and holistic lens. Psychological factors, including well-being, emotional regulation, happiness, and social isolation, emerged as key factors related to psychosocial risks, regardless of students’ professional future expectations. Organizational factors, including institutional values and the learning environment, also contributed to differences observed between students with high versus low future. Recognizing the interplay between individual and organizational factors supports the need for comprehensive, multilevel strategies to promote mental health and positive outlooks in higher education.

The findings provide strong valuable insights for higher education institutions, health professionals, and policymakers to develop and implement programs and interventions that support students academically, emotionally, and socially. By supporting students’ well-being and resilience, these initiatives may be associated with improved academic progression, adaptation to professional life and active contributions to society. Beyond advancing theoretical understanding, these results have clear practical implications, highlighting potential benefits both for students themselves and the socio-economic contexts in which they will participate.

## Data Availability

The raw data supporting the conclusions of this article will be made available by the authors, without undue reservation.
